# Clinical correlation between automated vision screening under cycloplegia and retinoscopy in young children

**DOI:** 10.5935/0004-2749.20230030

**Published:** 2023

**Authors:** Adriana Geremias, Nathalia Perussi Garcia, Caio Amadeo Silva Moreira, Guilherme Novoa Colombo Barboza, Marcello Novoa Colombo Barboza

**Affiliations:** 1 Hospital Oftalmológico Visão Laser, Santos, São Paulo, Brazil

**Keywords:** Refractive errors, Amblyopia, Strabismus, Refractometry, Retinoscopy, Erros de refração, Ambliopia, Estrabismo, Refratometria, Retinoscopia

## Abstract

**Purpose:**

To evaluate the clinical performance of the Spot Vision Screener and establish clinical correlations between automated screening and retinoscopy following induction of cycloplegia in preverbal children.

**Methods:**

In this prospective, cross-sectional study, children aged 6-36 months were evaluated using the Spot Vision Screener. A complete ophthalmologic examination, including cycloplegic refraction assessment, was performed, followed by repeat spot vision screening and retinoscopy in all cases to establish correlations regarding hypermetropia, myopia, and astigmatism following induction of induction cycloplegia.

**Results:**

The study included 185 children. The sensitivity of the automated screener after cycloplegia was 100% (95%CI: 85.18-100%), and specificity was 87.04% (95%CI: 80.87-91.79%). Positive and negative predictive values were 52.27% (42.36-62.01%) and 100%, respectively. Compared to retinoscopy, the Spot Vision Screener overestimated spherical values by 0.62 D (95%CI: 0.56-0.69) in the right eye and by 0.60 (95%CI: 0.54-0.66) in the left eye and cylindrical values by -0.38 D in the right eye (95%CI: -0.42-0.33) and by -0.39 D in the left eye (95%CI: -0.43-0.34). For overall spherical and cylindrical values, the difference was 0.61 D (95%CI: 0.57-0.65) and -0.38 D (95%CI: -0.41-0.35) in the left and right eyes, respectively.

**Conclusion:**

A substantial correlation was found between retinoscopy and objective data captured by the device. This shows that technology can be used in conjunction, reaching a more accurate diagnosis and identifying amblyopia risk factors as early as possible. Photoscreening may make a difference at the population level for early screening and intervention.

## Abstract (pt)

**Objetivo:**

Avaliar o desempenho clínico do *Spot Vision Screener* e estabelecer correlações clínicas entre a triagem automatizada e a retinoscopia após indução de cicloplegia em crianças pré-verbais.

**Métodos:**

Neste estudo transversal prospectivo, crianças de 6 a 36 meses foram avaliadas usando o *Spot Vision Screener*. O exame oftalmológico completo, incluindo refração cicloplégica, foi então realizado, seguido de repetição da triagem automatizada e retinoscopia em todos os casos, a fim de estabelecer correlações quanto à hipermetropia, miopia e astigmatismo após a indução de cicloplegia.

**Resultados:**

O estudo incluiu 185 crianças. A sensibilidade do dispositivo de triagem automática após cicloplegia foi de 100% (IC 95%: 85,18-100%) e a especificidade foi de 87,04% (IC 95%: 80,87-91,79%). Os valores preditivos positivos e negativos foram de 52,27% (42,36 - 62,01%) e 100%, respectivamente. Em comparação com a retinoscopia, o Spot Vision Screener superestimou os valores esféricos em 0,62 D (IC 95%: 0,56 - 0,69) no olho direito e em 0,60 (IC 95%: 0,54 - 0,66) no olho esquerdo e os valores cilíndricos em -0,38 D (IC 95%: -0,42 a -0,33) no olho direito e por -0,39 D (IC 95%: -0,43 a -0,34) no olho esquerdo. A diferença para os valores esféricos e cilíndricos de forma geral foi de 0,61 D (IC 95%: 0,57 - 0,65) e -0,38 D (IC 95%: -0,41 a -0,35), respectivamente.

**Conclusão:**

Foi encontrada correlação substancial entre a retinoscopia e os dados objetivos captados pelo dispositivo. Isso mostra que a tecnologia pode ser usada em conjunto, contribuindo para um diagnóstico mais preciso e identificando os fatores de risco de ambliopia o mais precocemente possível. A técnica automatizada pode fazer a diferença em nível populacional para triagem e intervenção precoce.

## INTRODUCTION

Amblyopia is the most common avoidable cause of vision loss in children, with a childhood prevalence of around 2%^(1,2,3)^. The prevalence of risk factors, such as high ametropia, anisometropia, and strabismus, is even higher, reaching as many as 15-20%^(4,5,6)^. Amblyopia is considered treatable up to 5 years of age, with a decline in the effectiveness of treatment after that^(7)^. Identifying these risk factors early represents a major challenge in primary healthcare settings^(8)^.

Although cycloplegic retinoscopy is the gold standard examination for the detection of ametropia and the cover test is the gold standard examination for strabismus in this age group, instrument-based screening may be helpful to the early detection of these precursors since retinoscopy is a difficult exam involving a long learning curve^(9)^. Developing countries where access to healthcare is limited may benefit from automated refraction systems in ophthalmologic screening. Furthermore, based on evidence, instrument-based screening is widely recommended by the American Academy of Pediatrics and the American Association for Pediatric Ophthalmology and Strabismus (AAPOS)^(7)^.

The Spot Vision Screener (Welch Allyn, Skaneateles Falls, NY, USA), an instrument developed to perform ophthalmologic screening, is a handheld device designed to easily and quickly screen patients aged six months or older^(10)^. It provides an automated evaluation and objective results for refractive errors, the presence of amblyopia risk factors, such as anisometropia and strabismus, and pupillary abnormalities. Device sensitivity and specificity have been reported as 81.8 to 89.8% and 70.4 to 88% in children 6-months to 16-years old^(10,11,12,13,14)^. Nevertheless, few studies have reported the accuracy of this method in children under three years of age. Forcina et al. evaluated 184 children aged 6-36 months without using cycloplegia or comparing the values of hypermetropia, myopia, and astigmatism obtained using the device with those found by an ophthalmologist during an examination^(13)^. Srinivasan et al. evaluated objective refractometry in 249 children aged 6-36 months^(15)^. Since screen capture was also performed without cycloplegia, a variation in accommodation might have occurred. Yakar evaluated the device performance before and after induction of cycloplegia; however, this study assessed 100 children aged 3-10 years, which is an older age group^(16)^.

The present study aimed to evaluate the clinical performance of the Spot Vision Screener as a screening device and establish the clinical correlation between automated screening and retinoscopy following induction of cycloplegia in children aged 6-36 months.

## METHODS

This prospective, cross-sectional study received approval from the institution’s internal review board under the reference CAAE: 25965019.1.0000.9367. All parents or guardians signed an informed consent form before the inclusion of their children in the study. The patients underwent a complete ophthalmic examination between September 2019 and May 2020. All the patients in the eligible age group (6-36 months) were included in the study.

### The Spot Vision Screener without cycloplegia

In all cases, the same medical student, specifically trained for the study, operated the Spot Vision Screener (firmware version 3.0.02.32, software version 3.0.05.00). At least three images were captured with the device for each patient to avoid errors resulting from poor positioning or inappropriate placement. Intermediate values were selected from results with low variability. When more than one measurement was taken, values tended to stabilize since the children became more cooperative as the examination proceeded. All examinations were performed in a room with low illumination in accordance with the manufacturer’s recommendations. The examiner selected the appropriate age group on the initial screen of the device and placed the device approximately one meter from a patient. Automated image capture then followed, with lights and sounds being produced by the device to engage the child. In two seconds, a report was issued on ocular alignment and binocular refraction, concluding with a statement that “*a complete ocular examination is recommended*” when the child needed to be submitted to a complete evaluation or “*screening complete*” when there was no need for pupil dilation or refraction testing. To determine report accuracy, all patients underwent supplementary evaluation.

### Complete ophthalmic examination

A pediatric ophthalmologist, masked to the results generated by the automated screener, examined the patients. Data were collected based on a detailed anamnesis to assess the patient’s complaints and ophthalmic exam. A unilateral cover test and an alternating cover test were performed to detect heterotropia and heterophoria. Next, cycloplegia was induced by instilling one drop of 1% cyclopentolate hydrochloride, followed by a second drop 5 minutes later. Reevaluation was performed 40 min after the instillation of the first drop.

#### Spot Vision Screener under cycloplegia

In this second stage, the same medical student captured three images again using the device under the same conditions established for the initial exam. The objective was to collect data following the induction of cycloplegia and compare those findings with the results obtained at retinoscopy.

#### Retinoscopy

Retinoscopy findings were used to determine ametropia objectively. The same pediatric ophthalmologist, who remained masked to the results previously obtained with the device, performed all the examinations.

### Analysis and interpretation of the results

The decision regarding when to correct ametropia was based on the refraction obtained at cycloplegic retinoscopy (the gold standard) and evaluation for the presence of eye misalignment by the pediatric ophthalmologist. The cutoff points were the same for the Spot Vision Screener and retinoscopy. The criteria used were those established in the 2013 AAPOS guidelines (Table 1)^(7)^.

**Table 1:** Amblyopia risk factors targeted with automated vision screening in preschoolers as established in the 2013 American Association for Pediatric Ophthalmology and Strabismus guidelines

Age (Months)	Refractive risk factor targets			
	Astigmatism	Hyperopia	Myopia	Anisometropia
12-30	>2.00 D	>4.5 D	>-3.50 D	>2.50 D
31-48	>2.00 D	>4.0 D	>-3.00 D	>2.00 D
>48	>1.50 D	>3.5 D	>-1.5 D	>1.50 D

### Sample size calculation

The study design specified the use of a Bland-Altman plot to compare the methods, with limits of agreement and their associated confidence intervals being calculated from the two measurements obtained for each patient. The two measurements would agree when the confidence intervals were within the boundaries set from the maximum allowable difference. For a power of 82.90% to detect agreement when the confidence level of agreement limits was 0.950, a sample of at least 180 subjects would be required. The maximum allowable difference in a standard deviation was 1.3, and the expected mean and standard deviation of the sample differences were 0.6 and 0.3, respectively.

### Statistical analysis

The kappa statistic was used to quantify the agreement between the Spot Vision Screener and the gold standard evaluation for strabismus and amblyopia risk factors. Alternative agreement coefficients were also used, as included in the Stata software program, version 14, under the command *kappaetc*^(17)^. The percentage of agreement and different ranges for kappa were calculated according to the following formulations: Brennan and Prediger, Cohen, Fleiss, Gwet’s AC, and Krippendorff’s alpha. An unweighted analysis was conducted for all the coefficients; therefore, the identity matrix described by Klein was used, and 95% confidence intervals (95%CI) were calculated.^(17)^ The coefficients were interpreted as follows: ≤0.00 = poor agreement; >0.00 to 0.20 = slight agreement; 0.21 to 0.40 = fair agreement; 0.41 to 0.60 = moderate agreement; 0.61 to 0.80 substantial agreement; and 0.81 to 1.00 = almost perfect agreement^(18)^. The sensitivity, specificity, and positive and negative predictive values of the Spot Vision Screener for the detection of amblyopia risk factors were calculated. With respect to the positive and negative predictive values, three different prevalence values were used: a) the prevalence of amblyopia risk factors based on the present sample; b) a value of 32.1% based on the findings of Forcina et al.^(13)^ and c) a value of 20% based on the results of Arnold^(19)^.

The Bland-Altman plot (difference versus mean) was used to compare the objective measurements obtained using the Spot Vision Screener with those obtained at retinoscopy. A smaller interval between the limits may be interpreted as better agreement, although the question of how small it should be depends on the clinical context^(20)^. The overall vertical dispersion of the points scatter reflects how closely the two measures agree, considering that for measures in perfect agreement, the plot would lie along the horizontal axis^(21)^.

## RESULTS

A total of 185 children (370 eyes) was included in this study. The median age of children was 21 months (mean 20 months, range 6-36 months). Of the total, 47% were female. Caucasian, black, and mixed-race children represented 75.7%, 1.1%, and 23.2%, respectively.

Seventeen of the 185 children (9.2%) had strabismus. There was 100% agreement between the Spot Vision Screener and ophthalmological examination for strabismus. Regarding the risk factors for amblyopia, table 2 shows the agreement between ophthalmological examination and the Spot Vision Screener and different ways of calculating the kappa. The agreement was almost perfect according to two calculation methods and substantial according to four, with the remaining alternative agreement coefficients showing substantial agreement.

**Table 2:** Kappa agreement and its variation between the ophthalmic examination and the Spot Vision Screener

	Coefficient	Standard error	t	p > [t]	95% Confidence interval	
Percent agreement	0.8865	0.0234	37.91	0.000	0.8403	0.9326
Brennan and Prediger	0.7730	0.0468	16.53	0.000	0.6807	0.8653
Cohen/ Conger’s kappa	0.6254	0.0716	8.74	0.000	0.4842	0.7666
Scott/Fleiss’ Pi	0.6173	0.0762	8.11	0.000	0.4670	0.7675
Gwet’s AC	0.8386	0.0364	23.04	0.000	0.7668	0.9104
Krippendorff’s alpha	0.6183	0.0762	8.12	0.000	0.4680	0.7685

**Figure 1:**
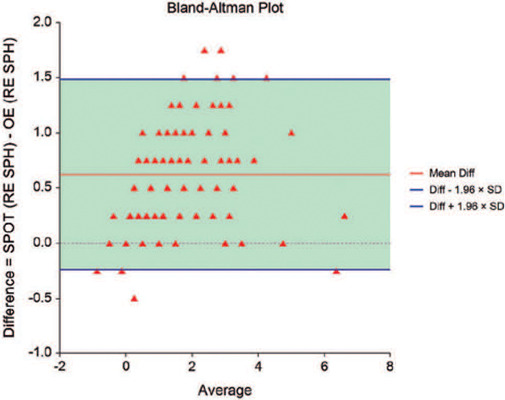
Bland-Altman plot for spherical correction. The vertical axis represents the diference for spherical correction in both eyes when values obtained at retinoscopy were subtracted from the values obtained using the Spot Vision Screener after cycloplegia.

**Figure 2:**
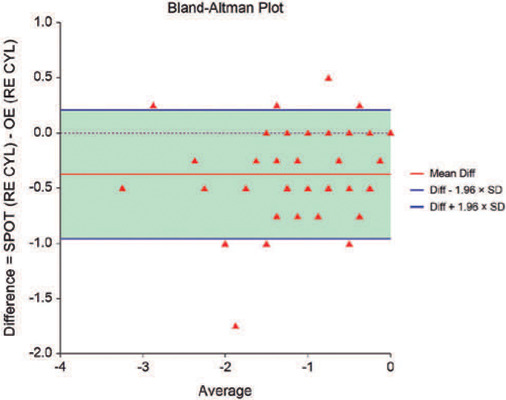
Bland-Altman plot for cylindrical correction. The vertical axis represents the diference for cylindrical correction in both eyes when values obtained at retinoscopy were subtracted from the values obtained using the Spot Vision Screener after cycloplegia.

The agreement between the Spot Vision Screener under cycloplegia and ophthalmological examination across the four objective measurements (spherical and cylindrical correction for the right and left eyes, respectively, and for both eyes together) is shown in figures 1 and 2. These graphs illustrate the difference in spherical and cylindrical correction for both eyes when the ophthalmological examination values were subtracted from the Spot Vision Screener values. There is a pattern of variability in the values, as shown by the standard deviation in the analysis.

Table 3 shows the Bland-Altman bias and limits of agreement, with the bias ranging from 0.38 to 0.62. Ideally, values close to zero would indicate no bias between the Spot Vision Screener under cycloplegia and the ophthalmological examination. The device overestimated hyperopia or underestimated myopia by 0.62 D in the right eye (95%CI: 0.56-0.69) and by 0.60 D in the left eye (95%CI: 0.54-0.66). For cylindrical corrections, the device also overestimated values by -0.38 D in the right eye (95%CI: -0.42-0.33) and by -0.39 in the left eye (95%CI: -0.43-0.34). Regarding the overall spherical and cylindrical values, there was a difference of 0.61 D (95%CI: 0.57-0.65) and -0.38 D (95%CI: -0.41-0.35), respectively.

**Table 3:** Bland-Altman bias and limits of agreement for ophthalmological parameters comparing ophthalmological examination and the Spot Vision Screener

Laterality	Measurement	Parameter	Count	Value	Standard deviation	95%CI	
Right Eye	Spherical correction in diopter	Bias	183	**0.62**	0.44	0.56	0.69
		Lower LoA	183	-0.24	0.06	-0.35	-0.13
		Upper LoA	183	1.48	0.06	1.37	1.59
Right Eye	Cylindrical correction	Bias	185	-**0.38**	0.30	-0.42	-0.33
		Lower LoA	185	-0.96	0.04	-1.03	-0.88
		Upper LoA	185	0.21	0.04	0.13	0.28
Left Eye	Spherical correction in diopter	Bias	184	**0.60**	0.44	0.54	0.66
		Lower LoA	184	-0.26	0.06	-0.37	-0.15
		Upper LoA	184	1.46	0.06	1.35	1.57
Left Eye	Cylindrical correction	Bias	185	-**0.39**	0.33	-0.43	-0.34
		Lower LoA	185	-1.02	0.04	-1.11	-0.94
		Upper LoA	185	0.25	0.04	0.17	0.33
Both eyes together	Spherical correction in diopter	Bias	367	**0.61**	0.43	0.57	0.65
		Lower LoA	367	-0.25	0.04	-0.32	-0.17
		Upper LoA	367	1.47	0.04	1.39	1.55
Both eyes together	Cylindrical correction	Bias	370	**-0.38**	0.31	-0.41	-0.35
		Lower LoA	370	-0.99	0.03	-1.05	-0.94
		Upper LoA	370	0.23	0.027	0.17	0.28

Bias= Difference between Spot Vision Screener and ophthalmological examination; LoA = Limit of Agreement

Table 4 shows the sensitivity, specificity, positive and negative predictive values of the Spot Vision Screener without cycloplegia used to detect amblyopia risk factors. Sensitivity, specificity, and positive and negative predictive values were 100% (95%CI: 85.18-100%), 87.04% (95%CI: 80.87-91.79%), and 52.27% and 100% (95%CI: 42.36-62.01), respectively. The positive and negative predictive values for a prevalence of 32.1% were 78.49% and 100% (95%CI: 70.99-84.46%). For a prevalence of 20%, the positive predictive value was 65.85% (95%CI: 56.41-74.19%), and the negative predictive value was 100%.

**Table 4:** Sensitivity, specifcity, and positive and negative predictive values (PPV and NPV) of the Spot Vision Screener for the detection of amblyopia risk factors prior to cycloplegia

	Value	95% Confidence interval
Sensitivity	100.00%	85.18%-100.00%
Specificity	87.04%	80.87%-91.79%
Positive likelihood ratio	7.71	5.18-11.50
Negative likelihood ratio	0.00	
Disease prevalence	12.43%	8.05%-18.07%
Positive predictive value	52.27%	42.36%-62.01%
Negative predictive value	100.00%	
Accuracy	88.65%	83.17%-92.83%

## DISCUSSION

As a screening system, photoscreening can be crucial, particularly in underdeveloped or developing countries where there are substantial inequalities in the distribution of infrastructure, meaning that not an entire population has equal access to healthcare. Screening for ametropia is vital if refractive errors and factors that could lead to amblyopia are to be identified in a timely manner since, in an age group such as this (6-36 months), children are not yet able to communicate and express their difficulties. Furthermore, cycloplegic retinoscopy, considered the gold standard, is a lengthy procedure that is examiner-dependent and associated with a long learning curve^(22,23)^. The present study evaluated agreement and reliability between automated screening and ophthalmic examination (the gold standard), with results showing substantial to almost perfect agreement.

Since its introduction in 2011, the validity of the Spot Vision Screener has been evaluated in children. Srinivasan et al. evaluated the performance of non-cycloplegic screening compared to cycloplegic retinoscopy in 249 children aged 6-36 months, with sensitivity and specificity of 66.7% (95%CI: 49.7-80.4) and 84.8% (95%CI: 79.1-89.3), respectively. The Spot Vision Screener was found to underestimate hypermetropia by 1.02 D (95%CI: 0.86-1.17). Conversely, it overestimated astigmatism by -0.52 D (95%CI: -0.43-0.62 D) compared to retinoscopy^(15)^. Gaiser et al. have recently evaluated the performance of the *Spot Vision Screener* without cycloplegia in 475 children aged 24-96 months. The sensitivity and specificity of the screener were 86.08% (95%CI: 76.45-92.84) and 90.15% (95%CI: 86.78-92.90), respectively. There was a variation of -1.34 D in spherical values with the Spot Vision Screener, while the values for astigmatism were overestimated by 0.48 D^(24)^. These data are also in agreement with the findings of Peterseim et al., who reported a variation of -1.35 D in spherical values, while astigmatism was overestimated by 0.36 D compared to cycloplegic retinoscopy^(14)^.

Since the previous studies compared the non-cycloplegic values of the Spot Vision Screener with retinoscopy, their results allow the screener to be evaluated merely as a screening instrument and not as an objective method due to the variability in accommodation, particularly when used in younger children^(25,26,27)^. The present study evaluated 185 children and compared the values obtained with the Spot Vision Screener under cycloplegia with those obtained at retinoscopy. In the evaluation of the spherical values following induction of cycloplegia, the Spot Vision Screener overestimated values by 0.62 D (95%CI: 0.56-0.69) in the right eye and 0.60 (95%CI: 0.54-0.66) in the left eye when compared to retinoscopy. Furthermore, there was a variation in the values for astigmatism of -0.38 D (95%CI: -0.42-0.33) in the right eye and -0.39 D (95%CI: -0.43-0.34) in the left eye. From a clinical point of view, these variations are completely acceptable in a prescription, particularly when age is considered, since this is the age group in which the accommodation tolerance is the greatest. According to the AAPOS guidelines, refractive errors of up to 4.50 D for hypermetropia, 3.50 D for myopia, 2.00 D for astigmatism, and up to 2.50 D for anisometropia can be tolerated without correction among children in the preverbal age group^(7)^. Therefore, the difference in the values obtained using the Spot Vision Screener and those obtained at retinoscopy following the induction of cycloplegia is considered acceptable in terms of adaptation. In fact, the device was not developed for use with cycloplegia; rather, its formula takes the patient’s expected accommodation (correction factor) into account. Consequently, the overestimated hypermetropia values were already expected since cycloplegia cancels out the tolerance of accommodation in these patients. Another aspect is that the values obtained were compared for the right and left eye separately. Unlike conventional refractometry, this is a binocular test. Nevertheless, the variation between the two eyes was minimal.

Yakar compared the performance of the Spot Vision Screener prior to and following the induction of cycloplegia in 100 older children (3-10 years). Results showed a sensitivity of 60.9% and specificity of 94.9% without cycloplegia and sensitivity of 85.3% and specificity of 87.4% following the induction of cycloplegia. The positive and negative predictive values were 85.7% and 90.4%, respectively, without cycloplegia and 63.6% and 95.8%, respectively, following the induction of cycloplegia^(16)^. Based on those results, the sensitivity of the screener was considered intermediate, and while specificity without cycloplegia was high, sensitivity increased significantly following the induction of cycloplegia^(16)^. The same strategy was used in the present study to analyze the clinical performance of the Spot Vision Screener; however, in a younger age group (<3 years). Sensitivity and specificity without cycloplegia were 100% (95%CI: 85.18-100%) and 87.04% (95%CI: 80.87-91.79%), respectively, equivalent to the results found among children older than 3 years.

Positive and negative predictive values are affected by the prevalence of the disease in the population. The assumption that sensitivity and specificity are fixed implies that the diagnostic performance test can be generalized for the population^(13^). For a prevalence of 32.1%, the positive and negative predictive values are 78.49% (95%CI: 70.99-84.46%) and 100%, respectively. For a prevalence of 20%, the positive predictive value is 65.85% (95%CI: 56.41-74.19%), and the negative predictive value is 100%. Despite the moderate positive predictive value found here, the negative predictive value found was excellent, which is a good indicator for a screening test.

There were two principal objectives in the present study: (1) to evaluate the clinical performance of the Spot Vision Screener; and (2) to evaluate the device as an autorefractor to determine hypermetropia, myopia, and astigmatism. Based on the sensitivity and specificity data shown here and fulfilling the first study objective, the screener proved to be an important tool in detecting the risk factors for amblyopia in the evaluated age group. The results show that the device can be used following cycloplegia as a supplementary test to complete ophthalmological examination with retinoscopy by discounting overestimated hypermetropia and astigmatism, thus, increasing the reliability of the clinical examination.

Qian et al. reported sensitivity and specificity of 94% and 80%, respectively, in a cohort of Chinese children aged 4-6 years without cycloplegia. Strabismus was also evaluated as an amblyopia risk factor, emphasizing the important agreement between the Spot Vision Screener and cover testing^(28)^. In relation to the detection of strabismus as a secondary finding of the study, the results obtained with the Spot Vision Screener agreed by 100% with the ophthalmic exam for the detection of heterotropia. Nevertheless, it is clear that binocular capture does not enable fusion break for the detection of heterophoria, and intermittent exotropia, for example, may not be identified at the time of examination. This is one of the limitations of the present study.

Other limitations must also be mentioned. One limitation refers to the determination of high ametropia. The device reaches spherical values of ±7.50 D and cylindrical values of ±3.00 D; however, the actual values in these cases were not determined. Nevertheless, only three eyes were excluded for these reasons. In addition, future visual acuity should be considered when analyzing the effectiveness of early intervention in the evaluated patients. Despite these limitations, the present study found a significant correlation between retinoscopy and the objective capture achieved by the device. This is the only study currently published in the literature in which the values achieved with the Spot Vision Screener under cycloplegia are evaluated in children under three years of age. Although no screening system can substitute clinical evaluation, technology can be used in conjunction, contributing toward reaching a more accurate diagnosis and identifying amblyopia risk factors as early as possible.

In the less affluent regions of the world, where access to healthcare can be limited, photoscreening may make a difference for population-based screening and early intervention, considering the difficulty involved in performing the exam in this age group. The present results confirm the effectiveness of the Spot Vision Screener, highlighting its importance as a tool for the early detection of risk factors that could lead to amblyopia and strabismus and as an additional test to quantitatively determine refractive errors in patients.

## References

[1] Multi-ethnic Pediatric Eye Disease Study Group (2008). Prevalence of amblyopia and strabismus in African American and
Hispanic children ages 6 to 72 months the multi-ethnic pediatric eye disease
study. Ophthalmology.

[2] Friedman DS, Repka MX, Katz J, Giordano L, Ibironke J, Hawse P (2009). Prevalence of amblyopia and strabismus in white and African
American children aged 6 through 71 months. The Baltimore Pediatric Eye
Disease Study. Ophthalmology.

[3] Pai AS, Rose KA, Leone JF, Sharbini S, Burlutsky G, Varma R (2012). Amblyopia prevalence and risk factors in Australian preschool
children. Ophthalmology.

[4] Borchert M, Tarczy-Hornoch K, Cotter SA, Liu N, Azen SP, Varma R, MEPEDS Group (2010). Anisometropia in Hispanic and african american infants and young
children the multi-ethnic pediatric eye disease study. Ophthalmology.

[5] Multi-Ethnic Pediatric Eye Disease Study Group (2010). Prevalence of myopia and hyperopia in 6- to 72-month-old african
american and Hispanic children: the multi-ethnic pediatric eye disease
study. Ophthalmology.

[6] Fozailoff A, Tarczy-Hornoch K, Cotter S, Wen G, Lin J, Borchert M, Writing Committee for the MEPEDS Study Group (2011). Prevalence of astigmatism in 6- to 72-month-old African American
and Hispanic children: the Multi-ethnic Pediatric Eye Disease
Study. Ophthalmology.

[7] Donahue SP, Arthur B, Neely DE, Arnold RW, Silbert D, Ruben JB, POS Vision Screening Committee (2013). Guidelines for automated preschool vision screening: a 10-year,
evidence-based update. J AAPOS.

[8] Ying GS, Maguire MG, Cyert LA, Ciner E, Quinn GE, Kulp MT, Vision in Preschoolers (VIP) Study Group (2014). Prevalence of vision disorders by racial and ethnic group among
children participating in head start. Ophthalmology.

[9] Alves MR, Schor P, Uras R, Veitzman S (2008). Optics, refraction and low vision.

[10] Silbert DI, Matta NS (2014). Performance of the Spot vision screener for the detection of
amblyopia risk factors in children. J AAPOS.

[11] Peterseim MM, Papa CE, Wilson ME, Davidson JD, Shtessel M, Husain M (2014). The effectiveness of the Spot Vision Screener in detecting
amblyopia risk factors. J AAPOS.

[12] Garry GA, Donahue SP (2014). Validation of Spot screening device for amblyopia risk
factors. J AAPOS.

[13] Forcina BD, Peterseim MM, Wilson ME, Cheeseman EW, Feldman S, Marzolf AL (2017). Performance of the Spot Vision Screener in children younger than
3 years of age. Am J Ophthalmol.

[14] Peterseim MM, Papa CE, Wilson ME, Cheeseman EW, Wolf BJ, Davidson JD (2014). Photoscreeners in the pediatric eye office: compared testability
and refractions on high-risk children. Am J Ophthalmol.

[15] Srinivasan G, Russo D, Taylor C, Guarino A, Tattersall P, Moore B (2019). Validity of the Spot Vision Screener in detecting vision
disorders in children 6 months to 36 months of age. J AAPOS.

[16] Yakar K (2019). Clinical performance of the Spot Vision Photo Screener before and
after induction of cycloplegia in children. J Ophthalmol.

[17] Klein D (2018). Implementing a general framework for assessing interrater
agreement in Stata. Stata J.

[18] Landis JR, Koch GG (1977). The measurement of observer agreement for categorical
data. Biometrics.

[19] Arnold RW (2013). Amblyopia risk factor prevalence. J Pediatr Ophthalmol Strabismus.

[20] Myles PS, Cui J (2007). Using the Bland-Altman method to measure agreement with repeated
measures. Br J Anaesth.

[21] Utley M, Gallivan S, Dixey J, Young A (1999). Correlation analysis versus Bland-Altman analysis: Comment on the
article by Genant et al. Arthritis Rheum.

[22] Barugel R, Touhami S, Samama S, Landre C, Busquet G, Vera L (2019). Evaluation of the Spot Vision Screener for children with limited
access to ocular health care. J AAPOS.

[23] Holmes JM, Lazar EL, Melia BM, Astle WF, Dagi LR, Donahue SP, Pediatric Eye Disease Investigator Group (2011). Effect of age on response to amblyopia treatment in
children. Arch Ophthalmol.

[24] Gaiser H, Moore B, Srinivasan G, Solaka N, He R (2020). Detection of amblyogenic refractive error using the Spot Vision
Screener in children. Optom Vis Sci.

[25] Fotedar R, Rochtchina E, Morgan I, Wang JJ, Mitchell P, Rose KA (2007). Necessity of cycloplegia for assessing refractive error in
12-year-old children: a population-based study. Am J Ophthalmol.

[26] Doherty SE, Doyle LA, McCullough SJ, Saunders KJ (2019). Comparison of retinoscopy results with and without 1%
cyclopentolate in school-aged children. Ophthalmic Physiol Opt.

[27] Yoo SG, Cho MJ, Kim US, Baek SH (2017). Cycloplegic refraction in hyperopic children: effectiveness of a
0.5% tropicamide and 0.5% phenylephrine addition to 1% cyclopentolate
regimen. Korean J Ophthalmol.

[28] Qian X, Li Y, Ding G, Li J, Lv H, Hua N (2019). Compared performance of Spot and SW800 photoscreeners on Chinese
children. Br J Ophthalmol.

